# So, and if it is not congenital adrenal hyperplasia? Addressing an undiagnosed case of genital ambiguity

**DOI:** 10.1186/s13052-022-01284-9

**Published:** 2022-06-10

**Authors:** Reinaldo Luna de Omena Filho, Reginaldo José Petroli, Fernanda Caroline Soardi, Débora de Paula Michelatto, Taís Nitsch Mazzola, Helena Fabbri-Scallet, Maricilda Palandi de Mello, Susane Vasconcelos Zanotti, Ida Cristina Gubert, Isabella Monlleo

**Affiliations:** 1 Maternal, Child and Adolescent Health Center, State University of Health Sciences of Alagoas, Postgraduate Program in Health Sciences of the Institute of Biological and Health Sciences of the Federal University of Alagoas, Maceió, Brazil; 2 Medical Genetics Sector, Faculty of Medicine, Federal University of Alagoas, Maceió, Brazil; 3 Laboratory of Human Molecular Genetics, Center of Molecular Biology and Genetic Engineering, State University of Campinas, Campinas, Brazil; 4 Institute of Psychology, Federal University of Alagoas, Maceió, Brazil; 5 Internal Review Board, Federal University of Parana, Curitiba, Brazil; 6 Clinical Genetics Service, Medical Genetics Sector, Faculty of Medicine, University Hospital, Federal University of Alagoas, Avenida Lourival Melo Mota, S/N, Tabuleiro 23 do Martins, 57072-970 Maceió, Alagoas, Brasil

**Keywords:** Rare disease, Ambiguous genitalia, HSD17B3 deficiency, Novel variant, Case report

## Abstract

**Background:**

The Congenital Adrenal Hyperplasia due to 21 hydroxylase deficiency is the most common cause of genital ambiguity in persons with XX sexual chromosomes. Genital ambiguity among persons with XY sexual chromosomes comprises diverse and rare etiologies. The deficiency of 17-beta-hydroxysteroid dehydrogenase type 3 enzyme (HSD17B3) is a rare autosomal recessive disorder due to functionally altered variants of the *HSD17B3* gene. In this disorder/difference of sex development, the conversion of androstenedione into testosterone is impaired. The appearance of external genitalia of 46,XY individuals varies from typically male to almost female.

**Case presentation:**

We report on a child presenting severe ambiguous genitalia. Due to access constraints, specialized care did not start until the child was 10 months old. Parents are consanguineous and were born in an area of high isonymy that is a cluster for rare recessive diseases. A new homozygous missense variant c.785G > T was found in exon 10 of the *HSD17B3* gene.

**Conclusions:**

Researchers-clinicians and researchers-researchers collaborative efforts to elucidate the genetic basis of this disease were critical since this etiologic investigation is not available through the public health system. This case exemplifies the families’ pilgrimage in cases of genital ambiguity due to a rare genetic condition. Recognizing the etiology was the baseline to provide information on prognosis and treatment options, and to shelter family and child doubts and hopes in order to better support their decisions.

**Supplementary Information:**

The online version contains supplementary material available at 10.1186/s13052-022-01284-9.

## Background

Rare diseases are always challenging for patients, their families and health professionals. When it comes to those that affect sexual development/differentiation, patients face other still complex vulnerabilities such as seeing himself/herself as different from the others, being targets of bullying in school age, and finding difficulties in understanding the situation [1].

The Congenital Adrenal Hyperplasia due to 21-hydroxylase deficiency is the most common cause of genital ambiguity in persons with XX sexual chromosomes. Genital ambiguity among persons with XY sexual chromosomes comprises diverse and rare etiologies [2].

The deficiency of 17β-hydroxysteroid dehydrogenase type 3 (HSD17B3; OMIM # 264300) is a good example. This 46,XY disorder/difference of sex development (DSD) is due to disruption of *HSD17B3* gene (OMIM * 605573) that impairs the conversion of androstenedione into testosterone mediated by the HSD17B3 enzyme. Inherited as an autosomal recessive condition, it shows a wide prevalence variation, ranging from 1:100–300 in Gaza Strip, where consanguineous marriages are frequent, to 1:147,000 in the Netherlands [3–7].

In cases of HSD17B3 deficiency, the external genitalia may range from male to almost female appearance including several degrees of ambiguity. Testes usually are in the inguinal canal or in the labioscrotal folds. Wolffian ducts are present, suggesting that low testosterone concentration is sufficient for internal male genitalia differentiation [5–8].

The recognition of genital ambiguity facilitates early diagnosis in childhood. On the other hand, individuals presenting minor genital changes and reared as girls may not be diagnosed until adolescence or adulthood. At this stage, primary amenorrhea and virilization of external genitalia may arise due to peripheral testosterone conversion [6, 9–11]. At any stage of life, this is a stressful situation that should be addressed carefully by an experienced multidisciplinary team in a patient-centered manner.

## Case presentation

**Fig. 1:**
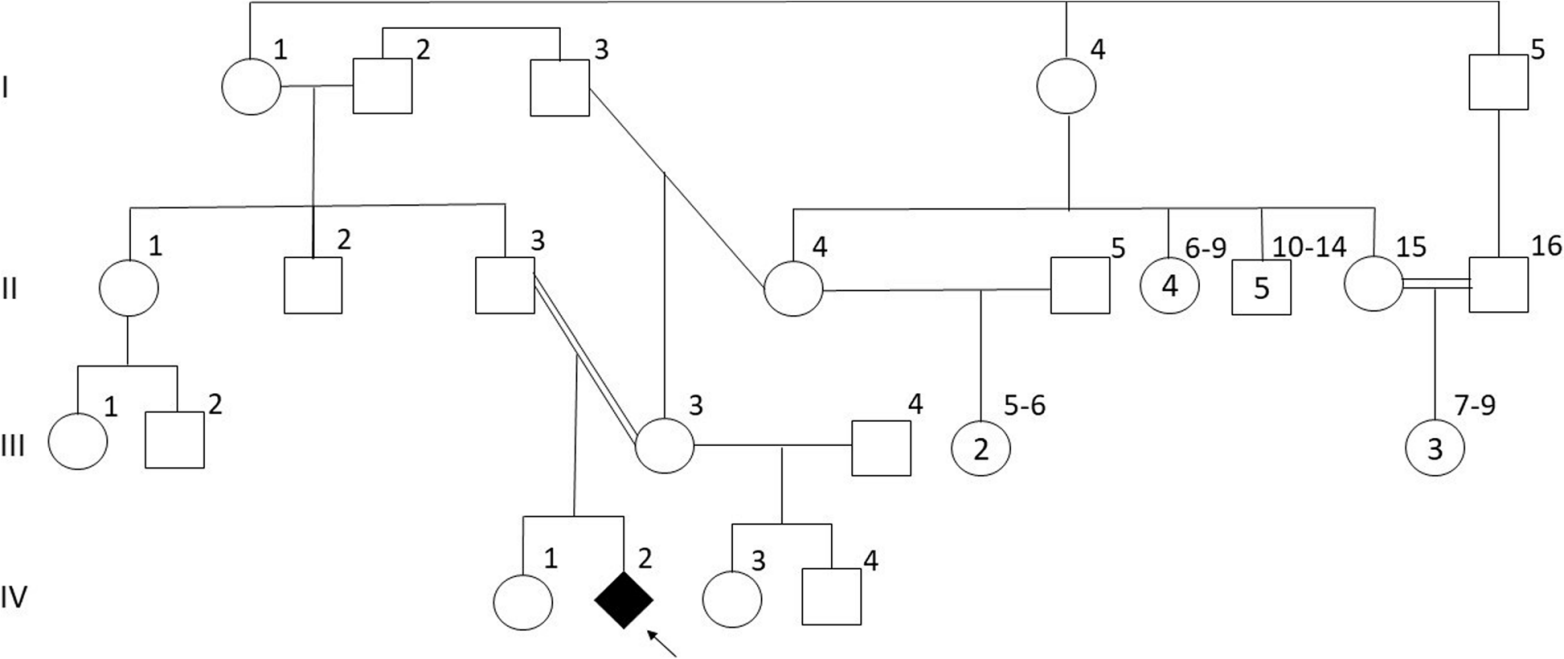
Family pedigree showing complex parental consanguinity

A 10-month-old child, assigned as female at birth, resident in the countryside of the Northeast region of Brazil, was referred to our team for genetic assessment due to genital ambiguity late recognized. Pregnancy and delivery were uneventful. Parents reported a clitoris enlargement and gonadal descent when the child was 30 days old, at the end of minipuberty. Afterward, they decided to rear him as a boy despite the maintenance of female legal sex. The patient is the second child in a consanguineous marriage (double first cousins once removed), with no recurrence in the family (Fig. 1).

**Fig. 2:**
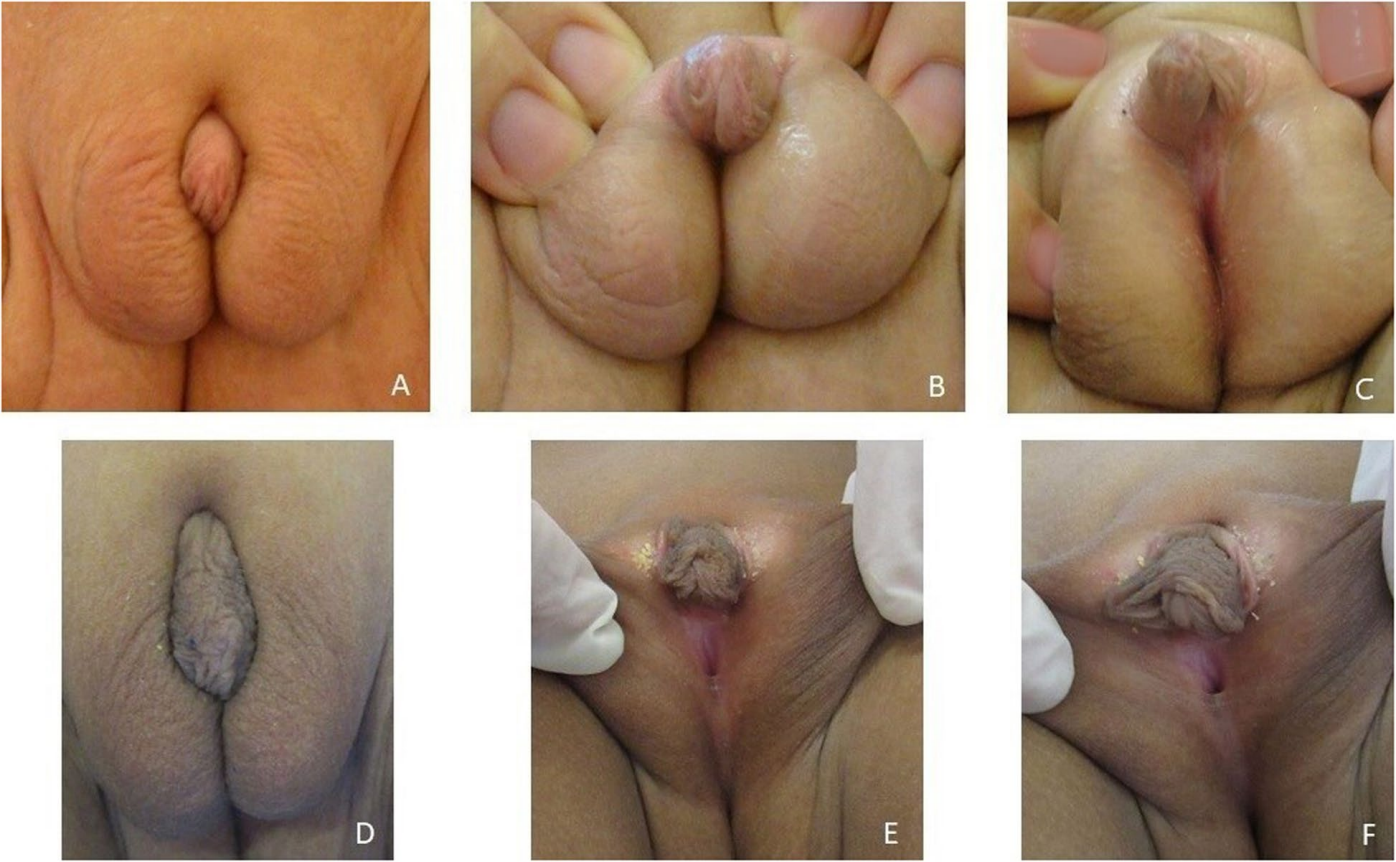
The external genitalia of our patient at 10 months (**A**, **B**, **C**) and at 6 years old (**D**, **E**, **F**)

Upon physical examination there was a phallus measuring 25 mm × 10 mm, palpable gonads at labioscrotal folds (1 cm^3^ left-sided and 0.5 cm^3^ right-sided), and an urogenital sinus with a perineal opening (Fig. 2). As these clinical findings indicate male prenatal under- instead of female hyper- virilization we assumed that Quigley type 4 [12] was the best classification for the ambiguous genitalia. The karyotype of peripheral lymphocytes was 46,XY. Basal FSH and LH levels were normal (1.32 mUl/mL and 0.34 mUI/mL, respectively) while those for testosterone were low (< 8 ng/dL). Abdominal ultrasound showed no Mullerian derivative structures.

The etiologic investigation included androgen receptor (*RA* – OMIM * 313700), steroid 5α-reductase 2 (*SRD5A2* – OMIM * 607306), nuclear receptor subfamily 5 group A member 1 (*NR5A1*– OMIM * 184757), and *HSD17B3* genes sequencing and predictive analyzes (Additional file 1).

Sequencing studies showed a novel homozygous c.785G > T nucleotide change in exon 10 of the *HSD17B3* gene (Additional file 2), which was inherited from both heterozygous parents. The c.785G > T substitution leads to the replacement of glycine by valine in residue 262 (p.Gly262Val).

Glycine 262 is a highly conserved residue (Additional file 3) and the comparison between them did not reveal changes on the protein internal contacts (Additional file 4). Four out of six predictive analyzes were compatible with a damaging missense variant and indicated that valine 262 destabilizes the protein structure (Additional file 5).

## Discussion and conclusion

### Clinical issues that impact on the family

The main cause of genital ambiguity is congenital adrenal hyperplasia, which can be lethal in the neonatal period if not diagnosed and timely treated. In this sense, neonatal screening represents a significant improvement in health care [2, 13].

On the other hand, genital ambiguity due to non-life-threatening etiologies is also an urgent situation because of the devastating impact it can have on the family and throughout the life of the person living with this clinical condition. Although these children may be recognized at birth, they are not usually diagnosed promptly, either because of the lack of knowledge of health professionals or the unavailability of genetic tests. The last may comprise diverse and complex molecular methods from peripheral karyotype to exome sequencing [14–19]. For many persons living in middle and low income countries, a simple karyotype may be unreachable.

The 46,XY DSD group is particularly challenging since different diseases share the same clinical features. This is the case of HSD17B3 deficiency that overlaps with other conditions that affect both the androgens synthesis or action. In the clinical setting, it is not always easy to establish a specific diagnosis since ambiguous genitalia with no Mullerian structures is a common feature in DSD, and basal hormonal probes are age-dependent. In cases of HSD17B3 deficiency, a testosterone/androstenedione ratio lower than 0.8 has shown a 100% diagnosis sensitivity in children up to six months old, illustrating the importance of early diagnosis. In any case, the molecular analysis of sex related developmental genes is a critical tool not only for diagnostic purposes but also for genetic counseling [6, 9–11].

It is noteworthy that although parents had noticed the genital ambiguity when the child was 30 days old, our patient was ten months old at first genetic assessment. The time elapsed illustrates the difficulty of accessing specialized care through the Brazilian Unified Health System (SUS). Some of the reasons that may explain this situation are the negligent genital examination of neonates, practitioners’ lack of knowledge on rare diseases, incoordination between the different levels of the SUS, shortfall of geneticists, and economical constraints [14].

The SUS assists around 80% of the Brazilian citizens which is an important achievement considering Brazil’s population of 214,4 millions, territory extension of 8.5 million Km^2^ and geographic and multiethnic diversity. Services are totally free of charge and based in a pyramid design with primary care in the basis and specialized tests, procedures and treatments in the very top. Since the implementation of SUS, 30 years ago, Brazil’s health indicators have substantially improved. As a consequence, chronic diseases, genetic ones included, became highly important [15].

Since the implementation of the national policy on rare diseases in 2014, the care of persons living with genetic disorders was gained a significant improvement. In the meanwhile, 17 rare diseases services, mostly located in the Southeast and South regions, were enabled to provide genetic assessment and counselling. However, the unavailability of genetic tests, including those for DSD, remains a bottleneck in the country as a whole [16–19]. Accessing these services is a challenge in a large country as Brazil especially for those with low income as the majority of Brazilian population. Accordingly, the distance between the residence and the specialized service was recently proposed as a global indicator of health care access [20].

Despite the lack of a rare disease service in the state of Alagoas, the genetic unity of the University Hospital from the Federal University of Alagoas has been providing genetic assessment and counselling since 2004. The unity has a multidisciplinary and voluntary team that provides open door care to patients with DSD. The genetic investigation is performed as a research protocol mainly in partnership with colleagues from the State University of Campinas. This collaboration eventually allowed us to investigate DSD etiology in the family herein reported.

Despite this achievement, it is important to note that three years have elapsed from the first consultation with us to the conclusion of genetic tests due to personnel and financial constraints. Meanwhile, the family gave up the follow up restarting it when the child was six years old. Currently, they are engaged with our team psychologist and continue rearing the child as a boy, as they have not decided on legal sex yet, which remains as female, and surgical and hormonal treatment.

### Molecular and genetic studies provision

The variant c.785G > T herein described causes the replacement of glycine by valine in the residue 262 of HSD17B3. It was checked against The Human Gene Mutation Database [21], ClinVar [22], Genome Aggregation Database [23], and Brazilian Genomic Variants [24] and it has never been reported before.

Glycine 262 is a conserved residue. Its side chains contain hydrogen, which provides conformational flexibility, while valine is a Cβ branched and hydrophobic amino acid with less conformational flexibility [25]. Therefore, the variant valine 262 can affect the protein due to the amino acid structure. The alignment between the wild-type and the variant protein (Additional file 4) shows that the amino acid change could affect the protein structure, although the internal contacts have not been affected. The scores of predictive algorithms PROVEAN, PolyPhen-2, Mutation Taster and Align GVGD were compatible with a damaging missense variation, while SIFT and MutPred2 showed a neutral effect (Additional file 5).

Upon these predictive analyzes, we hypothesize that the p.Gly262Val variant has led to a decrease in the HSD17B3 activity. As a consequence, testosterone synthesis was lowered to an insufficient rate to ensure our patients’ complete genital virilization. Thus, we suggest the p.Gly262Val variant is pathogenic. In vitro protein function studies should be carried out in order to validate these data.

The heterozygosis of this variant was found in both parents, who are double first cousins once removed as shown in Fig. 1. Consanguineous unions (those between persons with a common ancestor) and endogamy (union between persons belonging to the same community or social/ethnic group) are well-established risk factors for rare genetic conditions. These relationships have been studied for years in Brazil, with evidence of a significant impact in the Northeast region. By the end of 1990, the analysis of shared surnames (isonymy) arose as a powerful method to investigate migration, miscegenation, and isolation. Such population behaviors may be additional factors favoring the occurrence of rare diseases [26–30].

Our patient’s parents, as well as those of another case of HSD17B3 deficiency reported by the authors a few years ago [31], are consanguineous and come from an area with the highest rate of isonymy in Brazil [30].

The case presented here exemplifies how challenging the care of persons with rare DSD can be. From the patients’ perspective the vulnerabilities lie in the search for the correct and early diagnosis, the amount of analysis and all kinds of costs involved (time, emotional, and why not, financial) which may not be reached before a pilgrimage through specialists and health services [32, 33].

Additionally, patients and families need accurate information on diagnosis and prognosis to make complex decisions on rearing sex, and hormonal and surgical treatment [34–36]. These challenges are even higher in countries with abyssal inequities, such as Brazil, where patients’ pilgrimage is usually arduous.

In the reported case, the diagnosis of HSD17B3 deficiency was reached when the child was four-years-old as a result of a collaborative effort of researchers involved with DSD investigations. Nonetheless, the time elapsed has left its mark. The child is already six and is being reared as a boy, however, he is beginning to perceive differences between himself and other children. This situation is making the family’s suffering a continuous cycle and should be put on the table when discussing the wide impacts of undiagnosed rare diseases.

From a genetic viewpoint, the novel homozygous variant c.785G > T of the *HSD17B3* gene widen the molecular knowledge on this rare 46,XY DSD. Family pedigree alongside data on consanguinity and isonymy in Brazil corroborates the importance of the Northeast of the country as a cluster for autosomal recessive diseases.

## Supplementary Information

**Additional file 1.** Methods. Laboratory methods.

**Additional file 2.** Eletropherogram. Part of the electropherogram showing the homozygous change c.785G>T in exon 10 of the *HSD17B3* gene.

**Additional file 3.** Conservation analysis. Comparison between human and different mammalians HSD17B3 showing the conserved glycine 262 residue.

**Additional file 4.** HSD17B3 structure. Internal contacts established by HSD17B3 residue 262: A: wild type Gly262, B: variant Val262. C: 3-D structure modelled for wild-type HSD17B3 in blue and variant in red. D and E: zoom in the protein structure showing structural changes in the micro environment in residue 262, wild-type Gly represented in green and variant Val in Orange.

**Additional file 5.** Predictive analysis. Scores of predictive algorithms.

## Data Availability

The datasets generated and analyzed during the current study are not publicly available due individual privacy issues, however they may be available from the corresponding author on reasonable request.

## Abbreviations

DSD: Disorder/difference of sex development
HSD17B3: 17-Beta-hydroxysteroid dehydrogenase type 3
NR5A1: Nuclear receptor subfamily 5 group A member 1
RA: Androgen receptor
SRD5A2: Steroid 5α-reductase 2
SUS: Brazilian Unified Health System
