# Supplementary material for: So, and if it is not congenital adrenal hyperplasia? Addressing an undiagnosed case of genital ambiguity
Source: Ital J Pediatr. 2022 Jun 10;48:89. doi: 10.1186/s13052-022-01284-9 (PMC9188102; doi:10.1186/s13052-022-01284-9)
Supplement: Supplementary file 1 — Additional file 1. Methods. Laboratory methods. [file 13052_2022_1284_MOESM1_ESM.docx]

Methods

The *HSD17B3* gene (OMIM * 605573), located on 9q22.32, spans by 1,159 kb, comprises 11 exons and encodes a 310 amino acid protein. Sixty-seven pathogenic variants have already been described. Roughly, 66% of them are single-base changes, mainly missense/nonsense [1,2]

Genomic DNA was isolated from peripheral blood leukocytes using the proteinase K lysis and phenol/chloroform method. The eleven exons and exon-intron junctions of the *HSD17B3* gene were amplified by Polymerase Chain Reaction (PCR). Amplicons were purified and sequenced using both sense and antisense primers. The sequences obtained were compared to the *HSD17B3* reference sequence at the Ensembl database (ENSG00000130948) using Chromas and CLC Sequence Viewer v.6.6.2, both free software.

PROVEN, SIFT, PolyPhen-2, Mutation Taster, Align GVGD, and MutPred were used to perform predictive analyses of the novel variant identified. Molecular modeling was performed using the MODELLER web server program. The 17-beta-hydroxysteroid dehydrogenase X-ray crystal structure available at PDB (ID: 5FYD) was used as a template, and Kalign Multiple Sequence Alignment (free access) was used to compare human HSD17B3 and mammalian protein sequences.

**Reference**

1. HGMD® gene result [Internet]. [cited 2020 Jul 8]. Available from: http://www.hgmd.cf.ac.uk/ac/gene.php?gene=HSD17B3

2. Transcript: HSD17B3-202 (ENST00000375263.8) - Protein summary - Homo sapiens - Ensembl genome browser 100 [Internet]. [cited 2020 Jul 8]. Available from: http://www.ensembl.org/Homo_sapiens/Transcript/ProteinSummary?db=core;g=ENSG00000130948;r=9:96235306-96302176;t=ENST00000375263
