# Supplementary figures and images for: So, and if it is not congenital adrenal hyperplasia? Addressing an undiagnosed case of genital ambiguity
Source: Ital J Pediatr. 2022 Jun 10;48:89. doi: 10.1186/s13052-022-01284-9 (PMC9188102; doi:10.1186/s13052-022-01284-9)

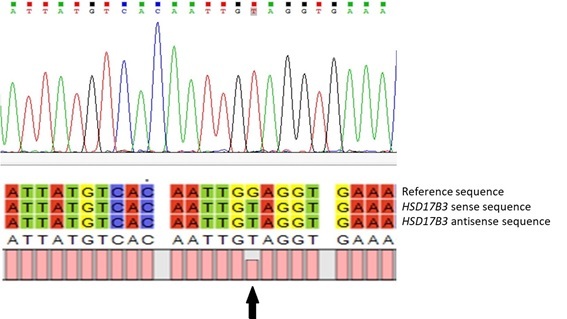

Supplement: Supplementary file 2 — Additional file 2. Eletropherogram. Part of the electropherogram showing the homozygous change c.785G>T in exon 10 of the HSD17B3 gene. [file 13052_2022_1284_MOESM2_ESM.jpg]

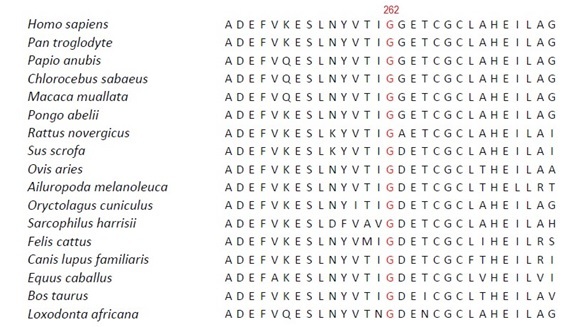

Supplement: Supplementary file 3 — Additional file 3. Conservation analysis. Comparison between human and different mammalians HSD17B3 showing the conserved glycine 262 residue. [file 13052_2022_1284_MOESM3_ESM.jpg]

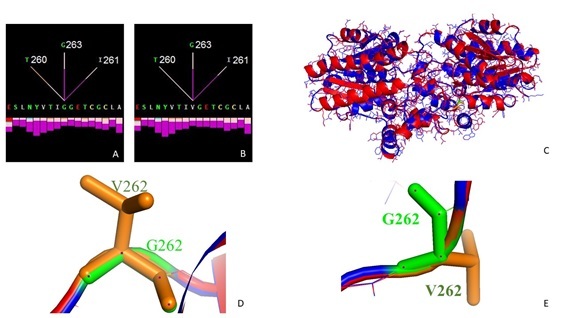

Supplement: Supplementary file 4 — Additional file 4. HSD17B3 structure. Internal contacts established by HSD17B3 residue 262: A: wild type Gly262, B: variant Val262. C: 3-D structure modelled for wild-type HSD17B3 in blue and variant in red. D and E: zoom in the protein structure showing structural changes in the micro environment in residue 262, wild-type Gly represented in green and variant Val in Orange. [file 13052_2022_1284_MOESM4_ESM.jpg]
