# Supplementary material for: So, and if it is not congenital adrenal hyperplasia? Addressing an undiagnosed case of genital ambiguity
Source: Ital J Pediatr. 2022 Jun 10;48:89. doi: 10.1186/s13052-022-01284-9 (PMC9188102; doi:10.1186/s13052-022-01284-9)
Supplement: Supplementary file 5 — Additional file 5. Predictive analysis. Scores of predictive algorithms. [file 13052_2022_1284_MOESM5_ESM.docx]

Scores of predictive algorithms

|  | **p.Gly262Val** | **Classification/Score** | |
| --- | --- | --- | --- |
| Algorithm | Result | Damaging | Neutral |
| *PROVEAN* | -4.230 | <-2.5 | >-2.5 |
| *SIFT* | 0,17 | <0,05 | >0,05 |
| *PolyPhen-2* | 1.0 | 1.0 | 0.0 |
| *Mutation Taster* | 109 | 215 | 0.0 |
| *Align GVGD* | C65 | C65 | C0 |
| *MutPred2* | 0.486 | 1.0 | 0.0 |
